# Association between clinical and MRI‐detected imaging findings for people with midfoot pain, a cross‐sectional study

**DOI:** 10.1002/jfa2.70019

**Published:** 2025-01-11

**Authors:** Jill Halstead, Carmen Martín‐Hervás, Elizabeth M. A. Hensor, Anne‐Maree Keenan, Philip G. Conaghan, Dennis McGonagle, John B. Arnold, Jennifer Jones, Anthony C. Redmond

**Affiliations:** ^1^ Leeds Community Healthcare NHS Trust Leeds UK; ^2^ Leeds Institute of Rheumatic and Musculoskeletal Medicine University of Leeds Leeds UK; ^3^ Radiology Department La Paz University Hospital Autonomous University of Madrid Madrid Spain; ^4^ Biomedical Research Networking Centre on Bioengineering Biomaterials and Nanomedicine Madrid Spain; ^5^ NIHR Leeds Biomedical Research Centre Leeds Teaching Hospitals Trust Leeds UK; ^6^ School of Healthcare University of Leeds Leeds UK; ^7^ Centre for Sport, Exercise and Osteoarthritis Research Versus Arthritis Nottingham/Leeds UK; ^8^ Alliance for Research in Exercise, Nutrition & Activity (ARENA) Allied Health & Human Performance University of South Australia Adelaide Australia

**Keywords:** FOAMRIS, foot, ligament, magnetic resonance imaging, midfoot, MRI, osteoarthritis, pain, tendon

## Abstract

**Background:**

Midfoot pain is common but poorly understood, with radiographs often indicating no anomalies. This study aimed to describe bone, joint and soft tissue changes and to explore associations between MRI‐detected abnormalities and clinical symptoms (pain and disability) in a group of adults with midfoot pain, but who were radiographically negative for osteoarthritis.

**Methods:**

Community‐based participants with midfoot pain underwent an MRI scan of one foot and scored semi‐quantitatively using the Foot OsteoArthritis MRI Score (FOAMRIS). Foot pain and disability were recorded using visual analog scales (VAS) and the Modified‐Manchester Foot Pain Disability Index (MMFPDI). Associations were assessed for continuous data using Spearman’s Rho, and for categorical data, a Wilcoxon signed rank test. Linear regression was used to explore the association between participant‐reported measures and MRI abnormalities, adjusted for age, sex and BMI.

**Results:**

Sixty‐one participants (70% female, mean age 48.5 years, median BMI 28.6 kg/m^2^) were included. Median VAS pain was 31/100 mm (IQR 21–47) and median disability was 30/48 (IQR 26–36). There was a moderate association between midfoot pain severity and the number of joints exhibiting joint space narrowing; adjusted results suggested 31% (95% confidence interval 3%–68%) worse VAS pain with each additional affected joint. Greater numbers of joints with cysts were associated with worse VAS pain [14% (0%–31%)] and disability [1.1 units (0–2.2)]. Effusion/synovitis was associated with MMFPDI pain. No other MRI abnormalities were associated with sex, body mass and foot pain/disability measures. Bone marrow lesions, joint space narrowing, cysts and osteophytes occurred more frequently with age. MRI abnormalities were common, particularly in the talo‐navicular joint, first and second cuneo‐metatarsal joints. Those with dorsal foot pain had more multi‐joint involvement, bone marrow lesions, joint space narrowing and cysts and for those with pain on midfoot movement, bone marrow lesions and cysts were reported.

**Conclusions:**

In people with midfoot pain, MRI‐detected features of osteoarthritis and soft‐tissue abnormalities were found, clustered in the medial and intermediate cuneiform joints. These features were more common with age but not associated with pain or disability measures. Younger people with dorsal midfoot pain exhibited early signs of bone and joint features of osteoarthritis and we recommend further imaging studies to determine the clinical and diagnostic significance.

## 1 BACKGROUND

One in five people in the UK over the age of 50 report midfoot pain [1, 2], but despite this being a common condition, the presentation and underlying causes are poorly understood. The most common finding is symptomatic radiographic osteoarthritis (OA), which affects one in eight people over the age of 50 in the UK and is strongly associated with physical disability and foot deformity [1, 3]. The midfoot includes all the structures from the neck of the talus to the mid‐shaft of the metatarsals; this includes 12 bones and joints with complex joint communications, eight long tendons, variable ligament orientations and a medial longitudinal arch that promotes shock attenuation and ambulation [4]. The midfoot is a complex region of the foot, where less is known about the causes of pain due to limitations of two‐dimensional radiography and deep structures beyond the scope of musculoskeletal ultrasound imaging [5]. To explore the bone, joint and soft‐tissues in three dimensions, magnetic resonance imaging (MRI) is considered the modality of choice.

Compared to other common sites of pain, like the hip and Knee [6], there is a lack of magnetic resonance imaging (MRI) research to explore the links with midfoot pain. The bulk of the MRI literature has concentrated on the characterisation of tibialis posterior tendon pathology [7], and more recently, there has been interest in early and established midfoot OA [8]. These studies show the medial midfoot joints (talo‐navicular and cuneiform joints) are susceptible to abnormalities and that this may be associated with flat foot deformity [8, 9, 10, 11]. Outside of these conditions, little is known about the value of clinical assessment, bone and joint structures and patterns of pathology identified on MRI in people with midfoot pain associated with weight‐bearing activities. This study sets out to describe patterns of MRI abnormalities and explores the associations between clinical assessment and midfoot symptoms.

## 2 METHODS

### 2.1 Aim and design

The aim of the study was to explore the association between clinical assessment and midfoot symptoms and describe patterns of MRI abnormalities. The MRI detected features were reported using the Foot Osteoarthritis Magnetic Resonance Imaging Score (FOAMRIS) in participants with medial midfoot pain who had concurrent radiographs, which had been reported to be normal. This was a cross‐section study design where baseline data was collected as part of an intervention study (trial registry ISRCTN 77862746) and radiographic midfoot OA was an exclusion criterion.

### 2.2 Participants

A sequential case series of 61 adults with medial midfoot pain was identified from consecutive community referrals from general practitioners to a community podiatry department from 2008 to 2011 (Leeds, Yorkshire, UK). Eligible participants were approached and provided written consent to be part of the study in accordance with the ethical approvals provided by Leeds West Ethics Committee (reference number: 09/H1305/10). Included participants had a pattern of midfoot pain associated with weight‐bearing activity that was present for 3 months or longer. All participants had undergone radiographic investigation by their GP upon referral or were sent for standard clinical radiography (anterior‐posterior and oblique views) prior to inclusion in the study. Potential participants were ineligible if they presented with clinical signs of midfoot OA (observed/palpable osteophytes and reduced joint‐motion) or were identified by the radiology report as having features of midfoot OA using standard clinical radiographs. Other exclusion criteria were contra‐indications for MRI; foot surgery in the last 12 months; foot pain typical of undiagnosed inflammatory arthritis (foot pain with diurnal variation, sudden onset with multiple joint pains, pain at rest and early morning stiffness of >30 min); neurogenic foot pain, or signs and symptoms of sensory abnormality (referred, diffuse, burning or tingling pains, allodynia and sensory loss); and a medical history of diabetes mellitus, peripheral arterial disease, kidney disease or organ transplantation.

### 2.3 Participant‐reported measures and assessments

Demographics measured were age, body mass index (BMI), as well as presence and activities reported with foot pain: walking, standing, climbing stairs, participating in sports/running and when wearing shoes. Foot pain severity (worst foot pain on the day) was measured using the 100‐mm visual analogue scale (VAS) [12, 13, 14] and foot pain‐related disability was measured using the modified Manchester foot pain and disability index (MMFPDI) [15, 16]. The MMFPDI contains 2 subscales (pain and function), with scores ranging from 0 to 21 (pain) and 0–28 (function) with higher scores indicating worse pain or disability. Participant pain maps identified the location of pain; drawn onto photos of the anterior, medial and lateral ankle; dorsal, medial and lateral midfoot; and dorsal medial and lateral forefoot. Assessment of the midfoot was recorded as pain with joint movement, this was reported at the talo‐navicular joints (TNJ), naviculo‐cuneiform joint (NCJ) and cuneo‐metatarsal joints (CMJ).

### 2.4 MRI acquisition

Once baseline measures were taken, the participant’s painful foot was scanned within one to 2 weeks using a Magnetom Verio (3T) MRI scanner (Siemens Medical Solutions, USA). If there was bilateral foot pain, the most painful was chosen, or if the pain was considered equal, a single foot was chosen using a test of first‐step limb dominance by asking the participant to turn in the opposite direction to the researcher and take a step forward, and walking initiation was taken to determine the leg dominance and study the foot. All images were acquired using an eight‐channel foot and ankle coil with the foot placed perpendicular to the ankle and magnetic field (β0). The following protocol and parameters were employed: T2‐weighted fat‐saturated sequence, TR:3000–3600 ms, TE:69, flip‐angle:155–160°, echo‐train length 8, 2 mm slices and 0.4 mm inter‐slice gap, matrix 256 × 256, FOV 150 × 150 mm in three planes; short‐tau inversion ratio sequence: TR:4500 ms, TE:31, NEX 2, TI:200, flip‐angle 150°, echo‐train length 11, 3 mm slices and 0.6 mm inter‐slice gap, matrix 320 × 256, FOV 150 × 150 mm in three planes; T1‐weighted high‐resolution spin echo TR:700 ms, TE:10, FS 3, flip‐angle: 90°, 1.2 mm slices and 1.32 mm inter‐slice gap, matrix 512 × 512, FOV 512 × 512 mm in the sagittal plane.

### 2.5 MRI scoring

Anonymised scans were analyzed using OsiriX 64bit Version 5.6 (OsiriX Foundation, Geneva, Switzerland). A single musculoskeletal specialist radiologist with 15 years’ experience read and scored the images using the Foot Osteoarthritis MRI Score (FOAMRIS) [17] that includes categorical, dichotomous or criterion‐based ranking (0–3) scores for joint space narrowing (JSN), osteophytes, joint effusion/synovitis, bone cysts, bone marrow lesion (BML), erosion and soft‐tissue features including tenosynovitis (tibialis anterior, extensor hallucis longus, extensor digitorum longus, peroneus brevis, peroneus longus, tibialis posterior, flexor hallucis longus and flexor digitorum longus) and abnormalities at the Lisfranc and inter‐tarsal ligament complex. The reliability of FOAMRIS showed intra‐reader agreement was generally good to excellent across the foot in joint features (JSN 0.94, osteophytes 0.94, effusion‐synovitis 0.62 and cysts 0.93), bone features (BML 0.89, erosion 0.78, BML‐entheses 0.79, BML sub‐tendon 0.75) and soft‐tissue features (tenosynovitis 0.90, ligaments 0.87) [17]. Inter‐reader agreement was lower for joint features (JSN 0.60, osteophytes 0.41, effusion‐synovitis 0.03) and cysts 0.65, bone features (BML 0.80, erosion 0.00, BML‐entheses 0.49, BML sub‐tendon −0.24) and soft‐tissue features (tenosynovitis 0.48, ligaments 0.50) [17]. The FOAMRIS Atlas was later created with representative images chosen for each of the severity scores for each plane and sequence to support the reporter to quantify the MRI score [18]. This Atlas was created using a total of 158 MRIs, of which, 35 were pain‐free and had no known OA, 69 had foot pain and 54 symptomatic radiographic foot OA (the full Atlas is available at: https://doi.org/10.5518/1568).

### 2.6 Statistical analysis

The descriptive assessment of the location and frequency of MRI abnormalities was identified in a numerical table, as well as the pictographic anatomical map to depict features that were reported in multiple joints/bones. Analysis included explorations of potential associations between (i) midfoot pain (VAS, MMFPDI) and demographic factors: age, sex and BMI and (ii) midfoot pain (VAS, MMFPDI) and disability index (MMFPDI) and MRI abnormality counts, which were tested using Spearman’s Rho (*r*). For categorical data, comparisons between (i) MRI abnormality and sex, (ii) MRI abnormality and midfoot joint movement and (iii) MRI abnormality and regions of pain were undertaken using a Wilcoxon signed rank test. A linear regression model was also developed to assess the relationship between midfoot pain (VAS) and midfoot abnormality counts, and this was adjusted for age, sex and BMI. A specific selection of midfoot abnormalities and cut‐off scores (graded 0–3) was pre‐determined by the research group as features that may be most likely to be clinically related to pain, disability and midfoot OA pathology. These included JSN >1, BML >0, metatarsal shaft BML >0, osteophytes >1, erosion >0, tenosynovitis >1 and effusion‐synovitis >0 and cyst >0 (scored present or absent). Analysis was undertaken using Stata (v13.1 StataCorp LP. 2013 Texas USA). The descriptive analysis of demographic data was undertaken using IBM (SPSS statistics v23) and interrogated for normality. In the case of normally distributed data, the mean and standard deviation (SD) were reported; if data were found to be non‐normally distributed, the median and inter‐quartile range (IQR) was reported.

## 3 RESULTS

The group consisted of the 61 participants, 43 (70%) were female sex (at birth) with a mean age of 48.5 years (SD 14.1, range 22–76). The median BMI was 28.6 kg/m^2^ (IQR 26–33, total range 21–43); 22 (36%) were classified as overweight (BMI >25) and 24 (39%) classified as obese (BMI >30). Participants tended to present with multiple co‐morbidities (median 2, IQR 1–3); most commonly OA (*n* = 21 34%, the majority reported pain at the knee and hip joints), followed by hypertension (*n* = 12, 20%), asthma (*n* = 9, 15%) and hypercholesterolaemia (*n* = 8, 13%).

### 3.1 Clinical presentation

Median midfoot pain severity was 31 mm on the VAS 100‐mm scale (IQR 21–47 mm) with a median pain duration of 10 months (IQR 6–22 months). Most of the group identified pain (using a pain map) in the dorsal (*n* = 44, 72%) and medial (*n* = 30, 49%) midfoot regions, followed by plantar (*n* = 16, 26%) and lateral midfoot (*n* = 5, 8%) regions. Single foot pain was present in 34 participants (56%) and bilateral foot pain was present in 27 participants (44%). The MMFPDI scores showed that the group experienced a median score of 30/48 for overall foot‐related pain and disability (IQR 26–36). The median foot function subscale score was 17/27 (IQR 14–21) and the median pain and appearance subscale scores were 13/21 (IQR 12–16). Pain on midfoot joint movement was present in 47 (77%) participants, with the highest proportion at the CMJs (*n* = 37,61%), followed by the NCJs (*n* = 16, 26%) and TNJs (*n* = 4, 7%). Midfoot pain when walking was reported by nearly all participants (*n* = 60, 98%), in addition to pain when standing (*n* = 25, 41%), climbing stairs (*n* = 15, 25%), participating in sports/running activity (*n* = 13, 21%) or when wearing shoes (*n* = 9.15%). Fewer participants reported pain while walking uphill (*n* = 6, 10%) or walking on uneven surfaces (*n* = 2, 3%).

### 3.2 MRI‐detected abnormalities (FOAMRIS)

**FIGURE 1:**
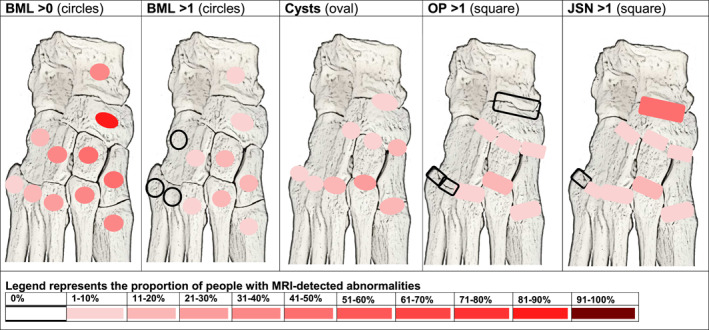
Person level anatomical map of bone marrow lesions (BML), cysts, osteophytes (OP) and joint space narrowing (JSN).

Using the semi‐quantitative imaging score, a high number of joints with MRI abnormalities was reported, which shows all participants had some pathology in the joints and tendons (effusion/synovitis, osteophytes and tenosynovitis). The most common feature was tenosynovitis reported in the entire group of 61 people (100%), followed by BMLs in 58 people (95%), osteophytes in 55 people (90%), JSN in 46 people (75%), cysts in 35 people (57%), ligament abnormalities in 33 people (54%) and bone erosion in 22 people (36%). By applying conservative cut‐off scores (grade >1), this reduced the proportion of MRI abnormalities for tenosynovitis to 27 people (44%), BMLs were reported in 32 people (52%), osteophytes 40 people (66%), JSN 13 people (26%) and erosions found in 5 (8%) (see Supplementary Tables). The most frequent locations affected in the midfoot were the TNJ, followed by the medial cuneiform and intermediate cuneiform bones and joints (see Table 1 and Figure 1).

**TABLE 1:** Number and frequency of MRI scores in the bones and joints of the midfoot per person reported using the FOAMRIS.

Anatomical locations	Bone		Joints		
	BML >0 *n* (%)	BML >1 *n* (%)	Cysts *n* (%)	OP >1 *n* (%)	JSN >1 *n* (%)
Talar neck	12 (20)	1 (2)			
Talo‐navicular joint			5 (8)	27 (44)	0 (0)
Navicular	31 (51)	6 (10)			
Navicular‐medial cuneiform joint			11 (18)	3 (5)	1 (2)
Medial cuneiform	28 (46)	7 (12)			
Navicular‐intermediate cuneiform joint			4 (7)	3 (5)	1 (2)
Intermediate cuneiform	25 (41)	11 (18)			
Navicular‐lateral cuneiform joint			4 (7)	1 (2)	1 (2)
Lateral cuneiform	19 (31)	6 (10)			
Medial cuneiform‐1^st^ metatarsal joint			10 (16)	4 (7)	1 (2)
1^st^ metatarsal proximal	19 (31)	1 (2)			
Intermediate cuneiform‐2^nd^ metatarsal joint			10 (16)	15 (25)	12 (20)
2^nd^ metatarsal proximal	24 (39)	9 (15)			
Lateral cuneiform‐3^rd^ metatarsal joint			3 (5)	7 (11)	1 (2)
3^rd^ metatarsal proximal	17 (28)	3 (5)			
Cuboid‐4^th^ metatarsal joint			4 (7)	3 (5)	0 (0)
4^th^ metatarsal proximal	12 (20)	0 (0)			
Cuboid‐5^th^ metatarsal joint			2 (3)	0 (0)	1 (2)
5^th^ metatarsal proximal	2 (3)	0 (0)			

*Note*: *n* = number, Bone marrow lesion = BML>1 (66%–100% of bone), osteophytes = OP > 1 (partial focal to full thickness loss), joint space narrowing = JSN ** calcaneus, cuboid and calcaneus‐cuboid joint not included see Supplementary Table S1.

**FIGURE 2:**
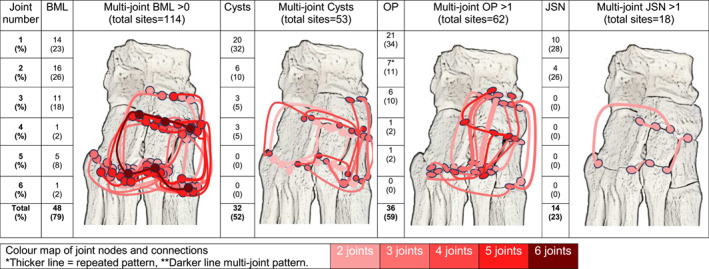
Person level map of multi‐joint MRI scored pathology in the midfoot (*n* = 61) for subchondral bone marrow lesions (BML), cysts, osteophytes (OP) and joint space narrowing (JSN).

Single sites and clusters of multi‐joint involvement were explored per feature (see Figure 2). The results showed the navicular was the most involved single bone, and the medial and intermediate cuneiforms were the bones most commonly involved in clusters. The medial CMJ was the most common single location for joint cysts (15% of cases), otherwise in two thirds of cases (63%) cysts presented in multiple joints with only one repeating pattern; two patients presented with cysts in the medial and intermediate CMJs and the medial NCJ.

**FIGURE 3:**
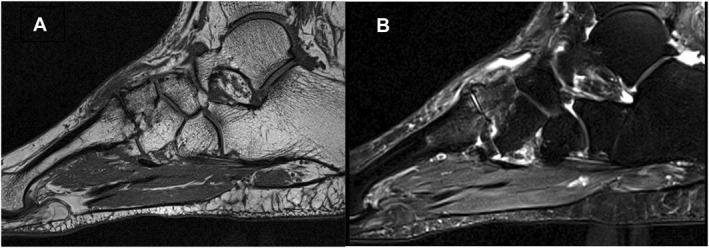
Sagittal plane T1 weighted (A) and STIR (B) MRI sequences show: Subchondral bone marrow oedema (grade 1) and dorsal osteophyte (grade 2) joint space narrowing (grade 2), bone erosion (grade1) at the intermediate cuneiform‐metatarsal joint. Small effusion at the tibio‐talar joint and talo‐navicular joint.

Joint space narrowing occurred in multiple joints for nearly three quarters of the cases (72%). The medial cuneiform bone was involved in 21% of multi‐joint cases (13/61), and the intermediate cuneiform bone was involved in 27% of multi‐joint cases (17/61) (see Figure 3).

Bone marrow lesions presented most commonly in multiple bones of the midfoot, only 20% presented in a single joint; the medial CMJ was the most frequent site (4 cases, 7%). There was a reoccurring pattern for BMLs in two and three bones clusters, the medial cuneiform bone was involved in 33% of cases (20/61 cases), and the intermediate cuneiform was involved in 38% of cases (23/61). There was no reoccurring pattern for groups of people who were scored with BMLs in four or six bones.

### 3.3 Associations between FOAMRIS and clinical measures

When the associations between pain and demographic factors were analyzed, pain severity VAS scores did not differ between males (median = 35 mm, IQR 26–59) and females (median = 30 mm, IQR 20–45) (*z* = 1.36, *p* = 0.174). There were also no associations between pain (VAS) and BMI (*r* = −0.01, *p* = 0.927) nor pain (VAS) and age (*r* = 0.08, *p* = 0.559). MMFPDI pain did not differ by sex (males median = 14, IQR 11–17; females median = 13, IQR 12–16; *z* = −0.03, *p* = 0.975) and was not associated with BMI (*r* = −0.03, *p* = 0.824); however, there was a weak negative correlation with age (*r* = −0.27, *p* = 0.036). MMFPDI disability did not differ by sex (males median = 16, IQR 14–20; females median = 18, IQR 13–21; *z* = −0.90, *p* = 0.366) and was not associated with BMI (*r* = −0.03, *p* = 0.824); however, there was a weak negative correlation with age (*r* = −0.27, *p* = 0.036).

While there were no differences in MRI abnormality counts in any of the joint and bones features between males and females (Table 2), there were substantive correlations with age (Table 3). The results suggested that older participants had more joints featuring cysts >0 (*r* = 0.51, *p* < 0.001), osteophytes graded >1 (*r* = 0.30, *p* = 0.02), JSN graded >1 (*r* = 0.31 *p* = 0.011), BML graded >0 (*r* = 0.45, *p* < 0.001) and erosions >0 (*r* = 0.45, *p* < 0.001).

**TABLE 2:** Differences between males and females regarding the MRI feature joint count.

Midfoot abnormality count	Male *n* = 18 median (IQR)	Female *n* = 43 median (IQR)	Wilcoxon *z*, *p* value
JSN >1	0 (0, 0)	0 (0, 0)	−0.11, *p* = 0.914
BML >0	2.5 (2, 6)	3 (2, 5)	−0.09, *p* = 0.930
Osteophyte >1	1 (0, 1)	1 (0, 2)	−0.78, *p* = 0.436
Cyst >0	1 (0, 2)	1 (0, 1)	0.44, *p* = 0.662
Erosion >0	0 (0, 1)	0 (0, 1)	0.27, *p* = 0.787
Effusion/synovitis >0	8 (6, 9)	9 (7, 10)	−2.12, *p* = 0.034
Met shaft BML >0	0 (0, 0)	0 (0, 0)	1.00, *p* = 0.316
Tenosynovitis >1	0 (0, 1)	0 (0, 1)	0.22, *p* = 0.829

*Note*: JSN = joint space narrowing, BML = bone marrow lesion, Met = Metatarsal.

**TABLE 3:** Correlations between FOAMRIS of midfoot abnormality counts and age and BMI.

Midfoot abnormality count	Age *r*, *p* value	BMI *r*, *p* value
JSN >1	0.32, *p* = 0.011*	0.23, *p* = 0.079
BML >0	0.45, *p* < 0.001*	0.16, *p* = 0.212
Osteophyte >1	0.30, *p* = 0.020*	0.02, *p* = 0.861
Cyst >0	0.51, *p* < 0.001*	0.19, *p* = 0.143
Erosion >0	0.45, *p* < 0.001*	0.13, *p* = 0.313
Effusion‐synovitis >0	0.15, *p* = 0.247	−0.34, *p* = 0.008*
Met. Shaft BML >0	0.04, *p* = 0.772	−0.22, *p* = 0.088
Tenosynovitis >1	0.09, *p* = 0.479	0.00, *p* = 0.977

*Note*: JSN = joint space narrowing, BML = bone marrow lesions, BMI = body mass index, Met = Metatarsal, * = *p* < 0.05.

With regards to BMI, there were no correlations with any of the joint or tendon abnormalities, other than a negative correlation with effusion/synovitis (*r* = −0.34, *p* = 0.008) (see Table 3).

We assessed midfoot joint movement by comparing MRI scores in participants with no movement pain to those with pain (see Supplementary Tables). Participants reporting pain with general midfoot joint movement (inversion and eversion) also had more bones scored with BMLs, and for those with specific CMJ movement, there was a greater number of participants with JSN, cysts, BML and erosions (see Supplementary Tables). Participants reporting dorsal pain also tended to have more bones with cysts, BMLs and erosions and JSN in dorsal bones and joints compared to patients reporting pain in other regions of the midfoot (see Supplementary Tables).

**FIGURE 4:**
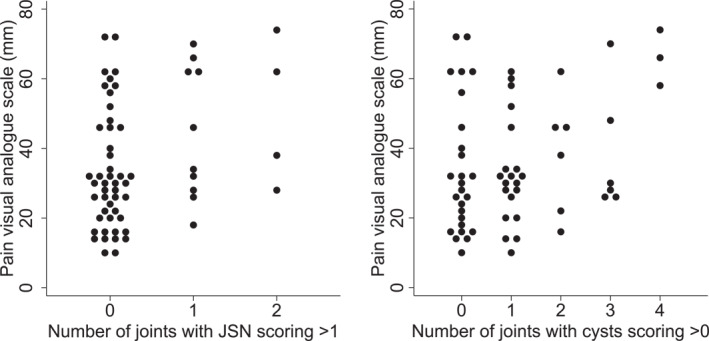
Association between pain and number of joints with JSN >1 and cysts >0.

Finally, linear regression was used to model the association between participant‐reported measures (VAS scores of worst midfoot pain on the day, MMFPDI pain and function subscales) and number of FOAMRIS‐rated abnormalities (Table 4). The VAS scores were natural log‐transformed to improve the distribution of the model residuals. Confounder‐adjusted estimates suggested weak‐to‐moderate positive associations between pain VAS and the numbers of joints with JSN (Figure 4) and joints with cysts (albeit at *p* = 0.057) although the 95% confidence intervals (CI) were wide. The CI for JSN indicated that pain scores might be between 3% and 68% higher with each additional midfoot joint with JSN score >1, while the CI for cysts ranged from 0% to 31%. For the MMFPDI pain subscale ,the 95% CI for effusion ranged from 0 to 0.9 additional units for each affected joint, all other CIs straddled 0. For the function subscale, the CI for cysts was between 0 and 2.2 (*p* = 0.057), remaining CIs straddled zero.

**TABLE 4:** Linear associations between midfoot abnormality counts and pain, adjusting for age, sex and BMI.

Midfoot abnormality count	% difference in per unit (95% CI), *p* value		
	VAS pain^a^	MMFPDI pain	MMFPDI function
JSN >1	31 (3, 68), *p* = 0.029*	0.6 (−0.8, 2.0), *p* = 0.396	0.0 (−2.1, 2.1), *p* = 0.985
BML >0	4 (−3, 11), *p* = 0.257	0.1 (−0.2, 0.5), *p* = 0.474	0.3 (−0.3, 0.8), *p* = 0.351
Osteophytes >1	−4 (−15, 9), *p* = 0.553	0.2 (−0.6, 0.9), *p* = 0.667	0.3 (−0.7, 1.4), *p* = 0.533
Cyst >0	14 (0, 31), *p* = 0.057	−0.6 (−1.4, 0.1), *p* = 0.113	1.1 (0.0, 2.2), *p* = 0.057
Erosion >0	9 (−11, 32), *p* = 0.408	−0.1 (−1.2, 0.9), *p* = 0.783	0.2 (−1.4, 1.8), *p* = 0.789
Effusion/synovitis >0	−1 (−9, 7), *p* = 0.803	0.5 (0.0, 0.9), *p* = 0.037	0.0 (−0.6, 0.7), *p* = 0.940
Metatarsal shaft BML >0	−9 (−36, 28), *p* = 0.569	0.5 (−1.4, 2.3), *p* = 0.618	1.1 (−1.7, 3.9), *p* = 0.430
Tenosynovitis >1	−5 (−24, 19), *p* = 0.675	−0.4 (−1.6, 0.8), *p* = 0.528	−1.1 (−2.9, 0.8), *p* = 0.244

^a^ For pain VAS the estimates represent the percentage difference in pain. JSN = joint space narrowing, BML = bone marrow lesion, MMFPDI = modified Manchester foot pain and disability index, VAS = visual analogue scale.

*p < 0.05.

## 4 DISCUSSION

The midfoot is a region with complex anatomy and multiple potential causes of pain. The bulk of the imaging studies has focused on osteoarthritis changes. Only one MRI study of the midfoot has explored the relationship with pain and or disability [8], with most other MRI studies focused on tibialis posterior tendon pathology [7].

Due to limited imaging studies in this field, this study set out to describe MRI findings in people with midfoot pain and without clinical or radiographic signs of midfoot OA. This study shows that in people with midfoot pain, there is a high frequency of low‐level joint and bone pathology and co‐existence of multiple pathologies with greater age. Low proportions of BMLs and joint effusion have been reported in healthy volunteer studies especially at the forefoot, and higher proportions have been shown to increase with age and inflammatory conditions [19, 20, 21]. This study adds to the current understanding of midfoot pain by presenting MRI findings and clinical assessments in a relatively younger group, using FOAMRIS to explore abnormalities of the joint, bone and soft tissues. The results show patterns of co‐existing MRI features that are clustered at the medial and intermediate CMJs: these 2 joints are also described as key sites of midfoot OA [8, 22] and represent a medial and central pattern of midfoot OA in people over 50 [23].

In this study, there was a limited association between the clinical presentation of midfoot pain and MRI features. These results have some similarity to a larger community study, which found a strong relationship with localised pain and radiographic OA but a weaker relationship with dorsal exostosis and joint movement [24]. In this study, people reporting dorsal midfoot pain and or painful joint movement (without palpable joint exostosis and normal X‐rays), a high proportion was reported to have patterns of subchondral BMLs and joint abnormalities typical of early OA. In addition, there was a moderate association found between the severity of foot pain and the number of joints reported with JSN. This would suggest features of OA should be considered in people with dorsal foot pain without X‐ray changes; however, the clinical significance of the midfoot features is yet to be determined. A high prevalence of OA features has been reported in a large UK community study [1] and retired Australian population study [22]. In the UK community study, 94% of people over 50 with midfoot pain were found to have a mild osteophyte score (Kellgren and Lawrence score = 1) on radiographs [1].

Unlike larger epidemiological studies of midfoot pain and osteoarthritis [1], there was no association found between midfoot pain and body weight or sex found in this study. This result was unexpected and may reflect a smaller, convenience sample and younger age range. When exploring pain measures and imaging features, disability was not associated with most of the bone and joint MRI scores, except for a small relationship between the number of cysts and disability. For pain severity, there was a weak association between the number of joints with JSN and to a lesser extent the number of bones with cysts. This is in agreement with MRI studies of the toe [25], midfoot [8] and knee [26], which showed higher rates of JSN in symptomatic OA groups. Previous knee studies have reported associations between pain MRI features like BML size and joint effusion/synovitis [27, 28], which was not found in the current study. In this exploratory study, grade one BMLs (up to 33% of the bone) were scored in 95% of the participants, which limited any associative analysis without a comparison control group. The frequency of BMLs and features of OA and cysts in this study was higher than those reported in an asymptomatic MRI study of the midfoot and hindfoot, which found these features increased after the age of 45 [20]. In a more recent study that included people with midfoot pain, radiographic midfoot OA and healthy controls; JSN, cysts, BMLs, and enthesopathy were weakly associated with symptoms, explaining between 9% and 17% of the variance in pain and 5% of the variance in foot‐related disability in patients with persistent midfoot pain and midfoot OA [8]. This work did not find the same associations between midfoot pain and imaging which requires further investigation.

This study set out to explore the association of MRI features and midfoot symptoms specifically in people who had already had a radiographic exclusion of established OA. The quality of the MRI scans of the foot joints, bones and soft tissues was likely assisted by imaging at 3T; however, the range of MRI sequences used in this study was limited, and further work is recommended to understand the optimal sequences for semi‐quantitative scoring in the foot. Limitations of this study include the moderate sample size, bias introduced with recruiting from community clinics and the absence of control participants with no foot pain to enable comparative analysis. We only adjusted our estimates for age, sex and BMI; there is likely to be additional confounding remaining, and inter‐relationships between MRI abnormalities were not explored here. Unfortunately, our findings in this convenience sample cannot be interpreted as causal effects; selecting only those with pain and without radiographic OA has the potential to induce bias. This series may have included some cases with radiographic midfoot OA, which may account for some of the findings. This is likely due to the absence of weight‐bearing X‐rays with a sagittal view as this increased diagnostic sensitivity [22]. The clinical screening attempted to ensure those with palpable or visible osteophytes were excluded; this was largely successful as only 4 joints (in four people) scored with a single grade 3 osteophyte. Larger studies with a control group, which collect a larger set of potential confounders, are recommended to explore the added utility of MRI scans in older people with midfoot pain compared to X‐ray alone.

Another factor to consider was the finding that imaging features were associated with pain severity measure (VAS) and not with pain‐related impairment subscale (MMFPDI), which counts severity as pain on some or most days. It is likely the difference between these two measures may be one reason for variation. In a study comparing the MFPDI pain subscale to a numerical rating scale, a moderate relationship (*r* = 0.5) between scores was reported that is contrary to this study, and the pain scale was shown to have lower internal consistency [29].

### 4.1 Recommendations for future studies

Further work exploring the clinical significance of MRI detected bone and joint changes in the midfoot is needed to explore how these changes (in terms of prevalence and size) may influence foot function and disability. To fully investigate the causal relationships between each of the pathologies and symptoms, proposed causal pathways would be required, identifying all potential confounders of these relationships and mapping any potential for implicit or explicit selection bias. It would be important to include people without midfoot pain and those with radiographic OA to ensure we obtained a sufficient spread of values for our exposures (the midfoot pathologies) and patient measures (symptoms) with appropriate re‐weighting of the data during analysis to account for any necessary oversampling of those with midfoot pain. This would require a large data set, as for each pathology (exposure), a different analysis model would be needed, adjusting for all relevant confounders (which would include any pathologies that tended to arise earlier in the pathway), but no mediators (i.e. any pathologies or other variables that tended to arise further on the pathway than the exposure). For instance, if joint space narrowing was proposed to occur prior to the development of BML, the model for the relationship between BML and symptoms should include JSN, but the model for JSN should not include BMLs.

The inter‐relationships between the different pathology types and their ordering of occurrence in the causal pathway are unclear at present, and this is particularly complex for a region such as the midfoot; in a cross‐sectional analysis, this would require several sensitivity analyses accounting for differing permutations. However, some of the visible pathologies, such as structural defects, are likely to be less prone to short‐term fluctuations than others, such as joint effusion and (teno)synovitis, which might help to hypothesise the likely order in which they would have achieved their observed scores, permitting cross‐sectional analysis. A longitudinal MRI scoring study with a cohort of people at risk of developing midfoot pain would be ideal in elucidating whether certain pathologies tended to occur before others.

## 5 CONCLUSION

In conclusion, this study suggests people with midfoot pain (with no clinical or radiographic signs of OA) are highly likely to have some joint abnormalities visible when assessing the midfoot with MRI, especially in people with older age. In younger people, with localised dorsal pain and pain with joint movement, features of OA are likely to be found on MRI scans and this could be a cause of pain. Bone, joint and soft tissue abnormalities occurred in multiple joints, with clusters occurring medially in the cuneiform and metatarsal bones. For most MRI features, our results did not suggest an association with pain severity, with the exception of the number of bones and joints reported with JSN and cysts in the dorsal region; however, confidence intervals were wide and included substantive effects for some other features. This study suggests that when evaluating midfoot pain, MRI features of OA in multiple joints are likely to be found and the clinical significance should be carefully considered.

## AUTHOR CONTRIBUTION

**Jill Halstead:** Conceptualisation; investigation; methodology; data curation; formal analysis; validation; writing ‐ original draft and writing ‐ review and editing. **Carmen Martín‐Hervás:** Conceptualisation; investigation; methodology; data curation; validation; writing ‐ original draft. **Elizabeth M. A. Hensor:** Data curation; methodology; formal analysis; validation; writing ‐ original draft; writing ‐ review and editing. **Anne‐Maree Keenan:** Funding acquisition; conceptualisation; methodology; investigation; supervision; writing ‐ original draft; writing ‐ review and editing. **Philip G. Conaghan:** Funding acquisition; conceptualisation; methodology; investigation; supervision; writing ‐ original draft; writing ‐ review and editing. **Dennis McGonagle:** Conceptualisation; methodology; investigation; supervision; writing ‐ original draft; writing ‐ review and editing. **John B. Arnold:** Data curation; methodology; formal analysis; validation; writing ‐ original draft; writing ‐ review and editing. **Jennifer Jones:** Data curation; investigation; validation; writing ‐ review and editing. **Anthony C. Redmond:** Funding acquisition; conceptualisation; methodology; investigation; validation; supervision; writing ‐ original draft; writing ‐ review and editing.

## CONFLICT OF INTEREST STATEMENT

All the authors declare that they have no competing interests.

## ETHICS STATEMENT

All participants provided informed written consent for the study. The ethical approval for the study was provided by Leeds West Ethics Committee (09/H1305/10) England UK.

## Supporting information

Supplementary Material

## Data Availability

The datasets used and/or analysed during the current study are available from the corresponding authors Dr. Jill Halstead [j.halstead‐rastrick@leeds.ac.uk] or Professor Anthony Redmond [A.Redmond@leeds.ac.uk] on reasonable request. For access to the Foot OA MRI Score and Atlas, please see https://doi.org/10.5518/1568 hosted by the University of Leeds repository.
