# Supplementary material for: Association between clinical and MRI‐detected imaging findings for people with midfoot pain, a cross‐sectional study
Source: J Foot Ankle Res. 2025 Jan 11;18(1):e70019. doi: 10.1002/jfa2.70019 (PMC11724207; doi:10.1002/jfa2.70019)
Supplement: Supplementary file 1 — Supplementary Material [file JFA2-18-e70019-s001.docx]

**Supplementary Data**

**Table S1** shows the frequencies of bone erosion and bone marrow lesions as scored by FOAMRIS per location

| Bone | Bone erosion >0  n (%) n (%) | | Bone erosion >1  n (%) n (%) | | BML >0  n (%) n (%) | | BML >1  n (%) n (%) | | |
| --- | --- | --- | --- | --- | --- | --- | --- | --- | --- |
| Metatarsal 1 proximal; distal | 2 (3); | 5/49 (10) | 0 (0); | 1/49 (2) | 19 (31); | 19/57 (33) | | 1 (2); | 6/57 (11) |
| Metatarsal 2 proximal; distal | 10 (16); | 3/49 (6) | 3 (5); | 1/49 (2) | 24 (39); | 2/47 (4) | | 9 (15); | 1/47 (2) |
| Metatarsal 3 proximal; distal | 1 (2); | 0/49 (0) | 0 (0); | 0/49 (0) | 17 (28); | 0/47 (0) | | 3 (5); | 0/47 (0) |
| Metatarsal 4 proximal; distal | 0 (0); | 0/49 (0) | 0 (0); | 0/49 (0) | 12 (20); | 0/58 (0) | | 0 (0); | 0/58 (0) |
| Metatarsal 5 proximal; distal | 0 (0); | 0/49 (0) | 0 (0); | 0/49 (0) | 2 (3); | 1/60 (2) | | 0 (0); | 0/60 (2) |
| Talar neck | 6 (10) |  | 1 (2) |  | 12 (20) |  | | 1 (2) |  |
| Calcaneus anterior | 1 (2) |  | 0 (0) |  | 12 (20) |  | | 1 (2) |  |
| Navicular | 1 (2) |  | 1 (2) |  | 31 (51) |  | | 6 (10) |  |
| Medial cuneiform | 3 (5) |  | 0 (0) |  | 28 (46) |  | | 7 (12) |  |
| Intermediate cuneiform | 5 (8) |  | 0 (0) |  | 25 (41) |  | | 11 (18) |  |
| Lateral cuneiform | 1 (2) |  | 0 (0) |  | 19 (31) |  | | 6 (10) |  |
| Cuboid | 0 (0) |  | 0 (0) |  | 17 (28) |  | | 0 (0) |  |
| no of participants* [total 61] (%) | 22 (36) |  | 5 (8) |  | 58 (95) |  | | 32 (52) |  |
| no of joints* [total 732] (%) | 30 (4) |  | 7 (1) |  | 218 (30) |  | | 45 (6) |  |
| Number of bones affected per patient* 0  1  2  3  >3 | 39 (64) |  | 56 (92) |  | 3 (5) |  | | 29 (48) |  |
|  | 15 (26) |  | 5 (8) |  | 7 (12) |  | | 22 (36) |  |
|  | 4 (7) |  | 0 (0) |  | 16 (26) |  | | 8 (13) |  |
|  | 2 (3) |  | 0 (0) |  | 8 (13) |  | | 1 (2) |  |
|  | 0 (0) |  | 0 (0) |  | 27 (44) |  | | 1 (2) |  |

BML = Bone marrow lesion *Excludes distal metatarsals which were not visualised in all patients - the midfoot was the main focus.

**Table S2** shows the frequencies of MRI joint features of FOAMRIS per location

| Joint | Effusion/  synovitis >0  n (%) | Cysts>0  n (%) | Osteophytes>0  n (%) | Osteophytes>1  n (%) | JSN>0  n ( %) | JSN>1  n (%) | Joint degeneration (Cy>0/ OP>0/ JSN>1)  n (%) |
| --- | --- | --- | --- | --- | --- | --- | --- |
| MTPJ 1 | 55/57 (96) | 19/57 (33) | 29/57 (51) | 6/57 (11) | 19/57 (33) | 5/57 (9) | 32 (56) |
| MTPJ 2 | 39/47 (83) | 1/47 (2) | 2/47 (4) | 1/47 (2) | 2/47 (4) | 2/47 (4) | 3 (6) |
| MTPJ 3 | 38/47 (81) | 0/47 (0) | 0/47 (0) | 0/47 (0) | 0/47 (0) | 0/47 (0) | 0 (0) |
| MTPJ 4 | 46/58 (79) | 1/58 (2) | 0/58 (0) | 0/58 (0) | 0/58 (0) | 0/58 (0) | 1 (2) |
| MTPJ 5 | 50/60 (83) | 1/60 (2) | 1/60 (2) | 0/60 (0) | 1/60 (2) | 0/60 (0) | 2 (3) |
| CMJ 1 | 57 (93) | 10 (16) | 29 (48) | 4 (7) | 14 (23) | 1 (2) | 31 (51) |
| CMJ 2 | 51 (84) | 10 (16) | 32 (52) | 15 (25) | 23 (38) | 12 (20) | 32 (52) |
| CMJ 3 | 46 (75) | 3 (5) | 24 (39) | 7 (11) | 1 (16) | 1 (2) | 24 (39) |
| CMJ 4 | 49 (80) | 4 (7) | 27 (44) | 3 (5) | 7 (11) | 0 (0) | 28 (46) |
| CMJ 5 | 50 (82) | 2 (3) | 11 (18) | 0 (0) | 1 (2) | 1 (2) | 12 (20) |
| NCJ - medial | 40 (66) | 11 (18) | 17 (28) | 3 (5) | 7 (11) | 1 (2) | 20 (33) |
| NCJ - intermediate | 24 (39) | 4 (7) | 14 (23) | 3 (5) | 6 (10) | 1 (2) | 15 (25) |
| NCJ - lateral | 24 (39) | 4 (7) | 9 (15) | 1 (2) | 5 (8) | 1 (2) | 9 (15) |
| TNJ | 56 (92) | 5 (8) | 55 (90) | 27 (44) | 4 (7) | 0 (0) | 55 (90) |
| Calcaneal-cuboid | 47 (77) | 4 (7) | 23 (38) | 6 (10) | 6 (10) | 0 (0) | 25 (41) |
| Subtalar | 58 (95) | 5 (8) | 1 (2) | 1 (2) | 1 (2) | 0 (0) | 6 (10) |
| Ankle | 52 (85) | 6 (10) | 5 (8) | 1 (2) | 11 (18) | 7 (11) | 11 (18) |
| no of participants* [total 61] (%) | 61 (100) | 35 (57) | 61 (100) | 40 (66) | 46 (75) | 16 (26) | 61 (100) |
| no of joints* [total 732] (%) | 554 (76) | 68 (9) | 247 (34) | 71 (10) | 95 (13) | 25 (3) | 268 (37) |
| Median no joints per person* (IQR) | 9 (8, 11) | 1 (0, 2) | 4 (3, 5) | 1 (0, 2) | 1 (1, 2) | 0 (0, 1) | 4 (3, 5) |

MTPJ = metatarsophalangeal joint, CMJ = Cuneiform/cuboid metatarsal joint, NCJ=Navicular–cuneiform joint, TNJ=Talo-navicular, JSN = joint space narrowing, IQR = inter quartile range, *excludes MTPJs as not visualised in all participants.

**Table S3** shows the frequencies of reported MRI tendon features of FOAMRIS per location

| **Tendon** | **Tenosynovitis (score>0)**  **n (%)** | **Tenosynovitis (score>1)**  **n (%)** |
| --- | --- | --- |
| Tibialis anterior | 23 (38) | 1 (2) |
| Extensor hallucis longus | 2 (3) | 0 (0) |
| Extensor digitorum longus | 5 (8) | 1 (2) |
| Peroneus brevis | 19 (31) | 0 (0) |
| Peroneus longus | 49 (80) | 5 (8) |
| Tibialis posterior | 50 (82) | 17 (28) |
| Flexor digitorum longus | 24 (39) | 1 (2) |
| Flexor hallucis longus | 18 (30) | 6 (10) |
| Total % of patients affected (n/61) | 61 (100) | 27 (44) |
| Total % of sites affected (n/488) | 190 (39) | 31 (6) |
| Number of sites affected per patient 0  1  2  3  4  >4 | - (0) | 34 (56) |
|  | 8 (13) | 24 (38) |
|  | 13 (21) | 4 (7) |
|  | 15 (25) | 0 |
|  | 15 (25) | 0 |
|  | 10 (16)  0 | 0  0 |

**Table S4** shows the frequencies of reported MRI ligament features of FOAMRIS per location

| **Ligament** | **Pathology present**  **n (%)** | **Joint associated** |
| --- | --- | --- |
| Lisfranc | 28 (46) | 2^nd^ Metatarsal medial cuneiform (n=26)  3^rd^ Metatarsal medial cuneiform (n=2) |
| Intertarsal | 10 (16) | 1^st^ to 2^nd^ cuneiform (n=1)  2^nd^ to 3^rd^ cuneiform (n=6)  2^nd^ lateral cuneiform-cuboid (n=1)  Lateral cuneiform-cuboid (n=1)  Lateral cuneiform-cuboid subluxation (n=1) |
| Total % of patients affected (n/61) | 33 (54) |  |
| Total % of sites affected (n/122) | 38 (31) |  |
| Number of sites affected per patient | 28 (46) |  |
| 1 | 28 (46) |  |
| 2 | 5 (8) |  |

**Table S5** Median abnormality counts in the midfoot according to whether the patient reported midfoot joint pain related to midfoot movement (inversion and eversion of the long midfoot axis)

| Midfoot abnormality count | No pain on midfoot movement n=14  Median count (IQR) | Pain on midfoot movement n=47  Median count (IQR) | Wilcoxon z, *p* value |
| --- | --- | --- | --- |
| JSN >1 | 0 (0, 0) | 0 (0, 1) | -1.63, p=0.104 |
| BML >0 | 2 (1, 3) | 4 (2, 6) | -2.12, p=0.034 |
| Osteophyte >1 | 0 (1, 1) | 1 (0, 2) | -1.00, p=0.319 |
| Cyst >0 | 0 (0, 1) | 1 (0, 2) | -1.79, p=0.074 |
| Erosion >0 | 0 (0, 0) | 0 (0, 1) | -1.45, p=0.149 |
| Effusion/synovitis >0 | 8 (7, 9) | 9 (7, 10) | -0.83, p=0.408 |
| Met shaft BML >0 | 0 (0, 0) | 0 (0, 0) | -0.08, p=0.933 |
| Tenosynovitis >1 | 0.5 (0, 1) | 0 (0, 1) | 0.21, p=0.838 |

JSN= joint space narrowing, BML= bone marrow lesion, Met=Metatarsal, IQR = inter quartile range

**Table S6:** Median abnormality counts in the cuneo-metatarsal joints and proximal metatarsals according to whether the patient reported cuneo-metatarsal joint pain related to movement of the joints dorsal and plantar in the sagittal plane.

| CMJ abnormality count | No pain on CMJ movement n=24  Median count (IQR) | Pain on CMJ movement n=37  Median count (IQR) | Wilcoxon z, *p* value |
| --- | --- | --- | --- |
| JSN >1 | 0 (0, 0) | 0 (0, 1) | -2.09, p=0.036 |
| BML >0 | 2 (1.5, 3) | 4 (2, 6) | -2.54, p=0.011 |
| Osteophyte >1 | 0 (1, 1) | 1 (0, 2) | -1.31, p=0.191 |
| Cyst >0 | 0 (0, 1) | 1 (0, 2) | -2.47, p=0.014 |
| Erosion >0 | 0 (0, 0) | 0 (0, 1) | -2.61, p=0.009 |
| Effusion/synovitis >0 | 8 (6, 9.5) | 9 (8, 9) | -1.21, p=0.228 |
| Met shaft BML >0 | 0 (0, 0) | 0 (0, 0) | -0.43, p=0.666 |

JSN= joint space narrowing, BML= bone marrow lesion, CMJ = cuneo-metatarsal joint, Met=Metatarsal, IQR = inter quartile range

**Table S7** Median abnormality counts in the dorsal region of the midfoot according to whether the patient reported pain in the dorsal region

| Dorsal abnormality count | No dorsal pain n=17  Median (IQR) | Dorsal pain n=44  Median (IQR) | Wilcoxon z, *p* value |
| --- | --- | --- | --- |
| JSN >1 | 0 (0, 0) | 0 (0, 1) | -2.50, p=0.013 |
| BML >0 | 0 (0, 1) | 2 (0.5, 3) | -3.37, p<0.001 |
| Osteophyte >1 | 0 (0, 0) | 0 (0, 1) | -0.98, p=0.328 |
| Cyst >0 | 0 (0, 0) | 0 (0, 1) | -2.26, p=0.024 |
| Erosion >0 | 0 (0, 0) | 0 (0, 1) | -2.39, p=0.017 |
| Effusion/synovitis >0 | 2 (2, 3) | 2 (2, 3) | 0.13, p=0.894 |
| Met shaft BML >0 | 0 (0, 0) | 0 (0, 0) | -0.65, p=0.513 |
| Tenosynovitis >1 | 1 (0, 1) | 0 (0, 1) | 0.87, p=0.387 |

JSN= joint space narrowing, BML= bone marrow lesion, Met=Metatarsal, IQR = inter quartile range
